# Understanding acute stress-mediated immunity in teleost fish

**DOI:** 10.1016/j.fsirep.2021.100010

**Published:** 2021-05-12

**Authors:** Huming Guo, Brian Dixon

**Affiliations:** Department of Biology, University of Waterloo, 200 University Ave W., Waterloo ON N2L 3G1, Canada

**Keywords:** Acute stress, Immunity, Fish, Teleost, Stress hormones, Catecholamines, Immunomodulation

## Abstract

The abilities and ways in which organisms respond to stress have long been demonstrated to affect the immune response of the organism. In mammalian studies, researchers have observed that chronic/long-term stress has a pronounced immunosuppressive effect, while studies in acute stress have demonstrated some immunoenhansive properties. These dynamics have been somewhat conserved in fish, as the effects of cortisol and chronic stress on the fish immune system are distinctly immunosuppressive, however, acute stress mediated immunomodulation is still poorly understood. This review explores the lesser studied non-cortisol stress hormones relevant to acute stress, and how they affect the immune response in Fish. Additionally, the effects of acute stress on various innate immune parameters and the regulation of immune related transcripts are discussed. Subsequently, this review attempts to establish the temporal transition between acute and chronic stress in the context of immune mediation. The conclusions of this review suggest that the modulating effects acute stress has on fish immunity is significantly different than that of chronic stress, yet more focused research must be conducted to further elucidate the mechanisms in greater detail.

## Highlights

• Stress/immune interactions are conserved between teleosts and mammals.
• In mammals chronic stress is immunosuppressive, acute stress can be immunoenhansive.
• In teleosts chronic stress is immunosuppressive, but effects of acute stress are not clear.
• Teleost acute stress has different effects than chronic stress but needs more study.

## 1 Introduction

Stress is a complex and conserved mechanism in higher level animals. It is a homeostatic regulatory mechanism that can affect many systems in the body either directly or indirectly. Externally, stress can be perceived from a source in the environment by sensory organs. Alternatively, it can be perceived internally through signaling of the hypothalamus. Various systems and pathways respond to stress hormones such as cortisol and catecholamines that are released during a stress response. Since “stress” is such a broad concept, the premise behind different stress-related studies varies drastically based on the focus of the researchers, although they study the same general concept. For example, differing factors such as duration, intensity, and type of stress can all significantly alter the requisite stress response in the organism.

In stress-immunity related studies, there is a comparative wealth of knowledge in the literature that focuses on the effects of long-term/chronic stress on immunity. More specifically, chronic stress and its main signaling mediator, cortisol, have traditionally been understood to have an overall suppressive effect on immunity [69,^84^,85]. This immunosuppressive effect of chronic stress has been proposed to have evolved from a need to conserve energy for processes critical for survival when the organism is struggling to maintain homeostasis [65]. Another proposed theory is that immunosuppression is the organism's attempt to attenuate autoimmune damage in times of stress [58]. In contrast, information on the effects of short-term/acute stress on immunity is relatively scarce, and this is doubly true in fish. While chronic stress has been extensively studied for its long-term implications for the fitness of wild animals and aquacultural impacts, acute stress has been relatively ignored. This is, perhaps, due to the difficulties with the logistics of experimental design, or lack of applicable impact causing lack of interest in the field. The goal of this review is to summarize acute stress mediated immunity in fish from various immunological angles and contextualize these results against other animal models.

## 2 Acute stress mediated immunity in mammals

Acute stress-mediated immunity has been an area of study in mammals for many decades. Thus, the level of understanding in mammalian literature is more advanced than the level of understanding in fish. However, with the progression of the field in fish, many of the recently elucidated mechanisms in fish are notably conserved with the mammalian models. With this in mind, it is worthwhile to first understand the state of this field in mammals, as a point of reference for fish-specific topics in later sections.

In both mammalian and fish models, chronic stress studies comprise the majority of stress-immunity related studies, this is especially true with earlier studies on the subject. This means that the effects of acute stress on immunity was a secondary focus that gained more traction relatively recently. Many of the earlier studies in mammalian acute stress-mediated immunity were related to the idea of immune tissue movement to the skin [9,31]. These studies observed redistribution of leukocytes from immune organs to peripheral tissues through the blood in the event of acute stress [20], [79]. Relatedly, this movement of granulocytes has also been observed following acute stress in the teleost model [4,56]. These mechanisms are understandable from an evolutionary perspective as acute stress is commonly associated with physical injury. Therefore, it would be advantageous for the organism's fitness if the immune system was organized in a way that can respond most efficiently to wounds and injuries. More specifically, subsets of immune cells such as dendritic cells, macrophages, neutrophils, NK cells, and T cells have been observed to infiltrate to peripheral tissues following acute stress [64], [78], [79]. These cellular trafficking mechanisms have been demonstrated to be signaled through immune relevant molecules such as interleukin-8 (IL-8), C-C motif chemokine ligand 2 (CCL2), interleukin-2 (IL-2), Interferon-gamma (IFN-γ), and tumor necrosis factor-alpha (TNF-α) [22,72]. Additionally, monocyte chemoattractant protein-1 (MCP-1), macrophage inflammatory protein-3 alpha (MIP-3α), interleukin-1 (IL-1), and interleukin-6 (IL-6) have also been implicated with the enhancement of immune activity in skin tissue [18].

Acute stress related redistribution of immune cells in mammals is governed by the release of stress hormones by the hypothalamus-pituitary-adrenal (HPA) axis. Traditionally, cortisol or corticosterone is the dominant immunosuppressive hormone throughout the course of chronic stress in both mammalian and fish models. However, some studies have shown that lower doses of corticosterone can enhance cell-mediated immunity, whereas both higher doses and chronic exposure to corticosterone will inhibit cell-mediated immunity [21]. This immunoenhancing effect of corticosterone is hypothesized to be caused by an upregulation of IL-2 receptor at a low dosage that is not present at a higher dosage [82]. Moreover, in acute stress, the immunoenhancement effects are signaled by a more complex combination of stress hormones, where cortisol/corticosterone does not play such a dominant role as it would in a chronic stress scenario. The trend observed from the culmination of many studies seems to suggest catecholamines, especially noradrenaline to be one of the most immunoenhancing stress hormones produced by the HPA axis [20], [64],72]. These relationships also appear to be conserved in fish (Section 3). Additionally, corticotropin releasing hormone (CRH) and adrenocorticotropic hormone (ACTH) have both demonstrated immunoenhancing activities as well [41,81]. Lastly, there may be additional noncanonical signaling factors at work outside of the typically studied stress hormones, but these understudied factors require further investigation to fully understand their roles in the complex stress-immune paradigm [77].

While much of the focus thus far has been on the enhancement of the innate response, studies have shown that acute stress is capable of enhancing the adaptive response as well [19,21]. In mice, it has been found that neuroendocrine stress before the administration of a vaccine results in better efficacy of memory response development and significantly increased immune response upon re-exposure to the original antigen [22]. In humans, trials with influenza and meningococcal vaccines found cohorts that were acutely stressed prior to vaccination produced higher antibody titers than the control cohorts [25,24]. Similar trends have been observed in fish models in regards to innate immunity and transcriptional analysis in immune-relevant tissues, albeit with less strength of evidence caused by contradictory results. In contrast, fish-related studies on acute stress and adaptive immunity are distinctly lacking.

Acute stress applied at various life stages of an animal has also been observed to affect immune responses in different and interesting ways for both mammals and fish [28]. Repeated acute stressors have been found to cause long-term development of glucocorticoid insensitivity in macrophages of mice, but not B cells [3,71]. In contrast, neonatal rats exposed to endotoxin resulted in increased glucocorticoid sensitivity as adults [67]. These contrasting cause-and-effect mechanisms of acute stress and glucocorticoid sensitivity have also been observed in humans, where a series of acute exercise and glucocorticoid studies demonstrated both increased and decreased sensitivity [23,70].

## 3 Effect of stress hormones on fish immunity

**Fig. 1:**
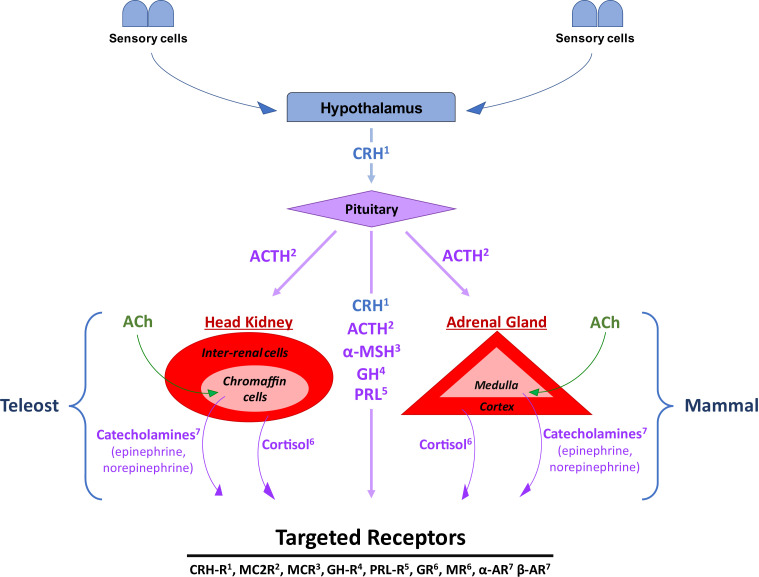
Graphical description of the differences and similarities in the tissues and signaling pathways in the stress response between fish and mammalian models. When stress is perceived by sensory cells in both fish and mammals, the signal is sent to the hypothalamus, which then releases corticotropin releasing hormone (CRH), the first major signaling hormone in this pathway. When CRH is detected by receptors in the pituitary, adrenocorticotropic hormone (ACTH) and α-Melanocyte-stimulating hormone (α-MSH) are released. The pituitary also releases growth hormones (GH) and prolactin (PRL). Additionally, ACTH stimulates the release of cortisol in the inter-renal cells of the fish head kidney, and the adrenal cortex of the mammalian adrenal gland. Alternatively, catecholamines are produced by the chromaffin cells of the fish head kidney and the adrenal medulla of the mammalian adrenal gland, the release of which is caused by signaling via acetylcholine (Ach). Superscripted values indicate the respective receptor type(s) for each hormone. The described hormones are, CRH-receptor (CRH-R), melanocortin 2 receptor (MC2R), melanocortin receptor (MCR), GH-receptor (GH-R), PRL-receptor (PRL-R), glucocorticoid receptor (GR), mineralocorticoid receptor (MR), α adrenergic receptors (α-AR), β adrenergic receptors (β-AR). Some receptor notations were generalized due to the lack of standardized notation between different fish species.

Although the general signaling structure of the stress response is conserved between mammals and fish, some notable differences have developed over time between the two groups. For instance, the adrenal glands in mammals are replaced by the analogous head kidney in fish. Hence, the stress modulating HPA axis in mammals is replaced by the hypothalamic-pituitary-interrenal axis (HPI axis) in fish (Fig. 1). In this system, the hypothalamic-pituitary interaction stays largely the same, secreting relevant stress signaling molecules like CRH and ACTH respectively, as well as other immunomodulating hormones such as growth hormone and prolactin. In the head kidney, the inner chromaffin cells are responsible for the secretion of the catecholamines, adrenaline and noradrenaline [38]. Whereas the outer interrenal tissue secretes cortisol [66].

As stated in the introduction, cortisol is the dominant stress hormone in the chronic stress response, whereas a combination of other stress hormones released from the HPI axis plays a bigger role alongside cortisol in an acute stress response. This sentiment was well elucidated by Ben Ammar et al. [8] who performed an interesting study where they exposed Atlantic salmon (*Salmo salar*) to a 10-minute acute stressor: the process of a fish passing through a power dam. Splenic mRNA expression of *lysozyme G, eosinophil peroxidase*, and *IgM* were increased post-stress. While plasma cortisol was not elevated in the stressed group compared to the non-stress group. A similar result was observed in an earlier paper, where temperature-related changes in cortisol levels did not decrease plasma IgM and IgM-secreting cell levels in common carp [63]. These results are in support of the ideas put forth by mammalian researchers that hormones and factors besides cortisol play a significant role in the acute stress mediation of immunity.

In head kidney studies, Castillo et al. [11] designed a holistic study on the effect of various stress hormones on *TNF-α, interleukin-1 beta (IL-1β), IL-6*, and *transforming growth factor beta 1 (TGF-β1)* transcript levels of gilthead seabream (*Sparus aurata*) head kidney cells. The results of this study observed that ACTH caused an increase in *TNF-α* and *IL-6* expression, a decrease in IL-1β expression, and an increase in *TGF-β1* expression. Adrenaline either did not affect transcript levels, or downregulated all assayed immune genes depending on the exposure length, while cortisol expectedly reduced expression levels of all cytokines examined. LPS + adrenaline and LPS + cortisol co-stimulation trials demonstrated attenuated immune responses compared to LPS only cohort in lowered IL-1β expression. While the LPS + ACTH cohort did not downregulate any assessed transcript, and *TNF-α* was found to be upregulated compared to LPS only [11]. In another set of *in vitro* experiments, [50] challenged maraena whitefish (*Coregonus maraena*) head kidney cultures with *Aeromonas* plus either cortisol, adrenaline, or noradrenaline. Of the three hormone treatments, cortisol had the most immunosuppressive effects, while adrenaline and noradrenaline were significantly less suppressive. Cortisol was also observed to decrease the expression of genes responsible for adrenergic and glucocorticoid receptors, while stimulation with noradrenaline increases these same parameters [50]. Likewise, another study revealed that adrenaline downregulated the production of radical oxygen species, pro-inflammatory cytokines, and chemokines in the phagocytes of common carp (*Cyprinus carpio*) [12]. From these results, it can be suggested that adrenaline and noradrenaline have distinctly different effects on the fish innate immune response. While adrenaline may present a more immunosuppressive influence, closer to the effects of cortisol, noradrenaline has a comparatively more enhancing/neutral effect on fish immunity. Relatedly, these conclusions were reverberated by findings in mammalian studies as well [64].

Other HPI axis hormones like ACTH and alpha-melanocyte-stimulating hormone (α-MSH) released by the pituitary have also demonstrated potential influence on fish immunity. *in vitro* study of phagocytes in rainbow trout (*Oncorhynchus mykiss*) have displayed increased phagocytic activity when exposed to α-MSH [34]. Another similar study in common carp observed both increased superoxide anion production and phagocytosis when stimulated with α-MSH [80]. To a similar effect, ACTH has been found to increase respiratory burst activity in rainbow trout [7], ACTH is also secreted by channel catfish (*Ictalurus punctatus*) leukocytes themselves in response to CRH presence, signaling possible paracrine and autocrine regulatory activities [1]. Combined with the discoveries of [11] above, there is substantial evidence for ACTH and α-MSH having immunoenhancing effects on fish innate immunity.

Lastly, other pituitary hormones such as growth hormone (GH) and prolactin (PRL) have demonstrated a variety of immunoenhancing effects, especially for innate immunity as reviewed by Yada and Tort [85]. In response to acute stress, researchers have observed a decrease in plasma GH levels [29,55], or no significant change in GH levels [2,^10^,^52^,^53^,68] following an acute stress challenge in several study systems. One 2017 study observed a slight increase in plasma GH levels and growth hormone receptor expression levels at 12-hours following acute handling and chasing stress [74]. Oppositely, a minimal decrease in GH receptor was observed in response to confinement stress [62]. Salinity is a dominant regulator of PRL in several fish species, in the presence of acute osmotic stress, animals will regulate PRL in an attempt to achieve osmotic homeostasis [10]. In response to other acute stressors, PRL release is generally found to be increased [52,86]. In summary, while these two pituitary hormones may be drastically regulated with specific stressors or other physiological conditions, most acute stressors do not seem to regulate these hormones with high magnitude compared to other stress hormones. Therefore, while these pituitary hormones have a relatively well-defined effect on immune response, it is still uncertain how relevant these effects are in the context of acute stress-mediated immunity, especially compared to the effects of catecholamines and glucocorticoids that are likely more central to the response.

## 4 The effect of acute stress on innate response parameters

Studies of innate immunity defense are relatively better understood in the context of stress-immunity in comparison to other areas of the field such as adaptive immunity. Most of the currently published data suggest that acute stress enhances the activities of these innate immune properties. One of the earlier studies was performed on the common dab (*Limanda limanda)*, where subjects were restrained in a dorsal position and manually rocked for 1-hour. This treatment resulted in an increase in blood phagocytes and a decrease in blood lymphocytes. Additionally, kidney and splenic phagocyte respiratory burst activity were also significantly stimulated in the stressed cohort [56]. Following acute crowding stress, an increase in peroxidase activity was observed in Gilthead Seabream [33]. Moreover, bactericidal activity has been assessed in conjunction with acute stressors by multiple research groups, and results of both increases and decreases have been reported [13,^36^,59]. Likewise, when researchers subjected Atlantic salmon to a 2-hour confinement trial, they discovered an increase in plasma bactericidal activity. However, they also observed a decrease in antibody production in the stressed cohorts following an *A.salmonicida* immunization, providing evidence for an acute stress influenced immune-enchancing effect that favors innate, but not adaptive responses [75].

Lysozyme activity has been repeatedly found to be elevated in response to acute stress, such as cold stress in Nile tilapia (*Oreochromis niloticus*) [46], and handling stress in rainbow trout [17]. Likewise, alternative complement pathway activity has been shown to increase with salinity stress in tilapia [39], and interestingly, these increases have been at least partially attributed to the action of catecholamines [88]. Lastly, Chen et al. subjected orange-spotted groupers (*Epinephelus coioides*) to varying levels of low salinity stress. When analyzed, total Leukocyte count, phagocytic activity, and respiratory activity were increased in the mildly stressed cohort but decreased in the severely stressed cohort [14]. These contrasting results among otherwise similar experimental systems demonstrate the stress-intensity specificity of stress-immunity interactions.

Some other contrasting results presenting immunosuppressive effects of acute stress on innate defense have also been published. A detailed study done by Costas et al. [15] exposed Senegalese sole (*Solea senegalensis*) to 3-minutes of air exposure stress, and found increased head-kidney leucocyte activity at 2- and 6-hours post-stress. Whereas plasma lysozyme and alternative complement pathway activity was decreased from 1- to 4-hours post stress compared to the non-stressed control cohorts [15]. Another recent study where common carp was subjected to a 3-hour ammonia challenge demonstrated a decrease in plasma lysozyme, complement, and bactericidal activity [59]. To a similar notion, Persian sturgeon (*Acipenser persicus*) subjected to a 30-minute crowding stress did not present any changes in plasma lysozyme levels despite an observed increase in plasma cortisol [35]. It is possible the discrepancies in these related studies are at least partially caused by the difference in species [5] and stressor parameters. Holistically, the presently available information suggests a possible increase in innate immune activity and function in specific stress conditions and animal species, but also demonstrated decreases in innate immune parameters in other study systems.

## 5 Differential immune gene expression in response to acute stress

Transcriptional analysis of immune-related genes is another commonly applied method used to investigate the effect of acute stress on fish immunity. As such, the head kidney has been one of the most heavily studied organs regarding stress-immunity induced transcriptional regulation. To date, substantial evidence across multiple papers have associated acute stress with an upregulation of pro-immunity transcripts. A conclusive study conducted by Hoseini et al. [37] subjected common carp to transportation stress for 4-hours at both low and high stocking densities. Immediately after transport, head kidney *TNF-α, IL-1β,* and *IL-8* expression levels were increased in both low- and high-density groups when compared to the non-stressed controls. Furthermore, the high stocking density group expressed significantly higher transcript levels than that of the low-density group for all three genes. Similar results were also presented by Metz et al. [52] who found increased expression of *IL-1β* and *IL-1β* receptors in the head kidney of Common carp after an acute restraint stress trial. Both hypo- and hyperosmotic acute stress *in vitro* conditions significantly upregulated *TNF-α, IL-1β, IL-6* and *suppressor of cytokine signaling 1* (*SOCS-1*) expression levels in the spotted scat (*Scatophagus argus*). In both stress conditions, expression levels of all four transcripts peaked at either 3- or 6-hours post-stress, and gradually declined towards the 15-hour timepoint [73].

In another study, [30] exposed Atlantic salmon to a shorter 15 s acute stressor and thereafter separated head kidney macrophages for *in vitro* analysis. The acute stressed cohort expressed higher levels of constitutive *IL-1β* in the head kidney macrophage cultures than the non-stressed cohorts. However, in cohorts challenged with LPS, the acutely stressed cohort expressed lower levels of head kidney macrophage *IL-1β* than the LPS-non-stressed cohort. These results by Fast et al. [30] highlight the perspective that basal level increases in transcript expression do not always translate to increased protection under pathogenic challenges.

In contrast to the above upregulation, Machado et al. [47] did not observe increases in head kidney expression of pro-immunity genes in Senegalese sole in response to water acidification, but a significant decrease in *interleukin-10 (IL-10)* was reported, suggesting a possible anti-inflammatory state. Likewise, acute anesthetic stress in common carp resulted in decreased head kidney *TNF-α* and *IL-1β* transcripts and returned to pre-stress levels after 24-hours, this timeframe understandably coincided with a respective increase and decrease in plasma cortisol concentrations [36].

Aside from the head kidney, other tissues have also displayed differential transcript regulation in response to acute stress. Liver expression levels of *IL-8, TNF-α, IL-1β, major histocompatibility complex I (MHC I),* and *major histocompatibility complex II (MHC II)* have all been found to be upregulated in various capacities in a myriad of acute stress conditions [49,^57^,^83^,89]. There is also evidence of upregulation of *TNF-α* and *IL-1β* expression levels in other non-primary immune organs like muscle, intestines, and brain tissues [89]. In these analyses of transcript expression data, results need to be contextualized through tissue and organ type. As a pro-inflammatory profile in the spleen caused by acute stress may be immunoenhancing, whereas the same profile caused by chronic stress in brain tissue is most likely deleterious [45].

A study designed to assess the effects of acute stress on antigen uptake during vaccination of olive flounder (*Paralichthys olivaceus*) observed increased antigen uptake of inactivated *Edwardsiella tarda* after acute hyperosmotic exposure. Additionally, transcripts of *MHC Iα, MHC IIα, cluster of differentiation 4–1* (*CD4–1*), and *cluster of differentiation 8α* (*CD8α*) were upregulated in spleen, head kidney, and liver tissues [32]. Khansari et al. [42] published a study analyzing the effects of acute-stress and vaccination on the transcriptomic response of various mucosal tissues, with an additional factor by contrasting between results from a freshwater fish (rainbow trout) and a marine fish (gilthead seabream). The animals were treated to 1-minute air exposure, a *Vibrio anguillarum* vaccine, or a combination of both treatments. Overall, acute air exposure caused an immune-enhanced transcription profile in both organisms. Additionally, the stressor and tissue types in which immunoenhancement effects were observed were notably different between the two species. The authors suggested adaptation to different environmental factors as a key reason for the disparity observed between the species. Other studies conducted by the same group echoed the idea of species related disparity in stress-mediated immunomodulation ([44,43]). Holistically, the results referenced in the above sections of all transcriptional regulatory changes has been summarized in (Table 1.)

**Table 1:** Summary of transcriptional regulation changes in various fish species subjected to different acute stress regimes.

Author	Species	Stress type	Stress duration	Tissue type	Effect on immune transcripts
Kirsten et al.	Danio rerio	air exposure	60 s	brain	↑TNF-α, IL-1β, IL-10
Zhao et al.	Schizothorax prenanti	hypoxia	24 h	brain, gills, liver, muscle	↑TNF-α, IL-1β
Hoseini et al.	Cyprinus carpio	transportation	4 h	head kidney	↑TNF-α, IL-1β, IL-8
Metz et al.	Cyprinus carpio	restraint	24 h	head kidney	↑IL-1β, IL-1β-r
Fast et al.,	Salmo salar	handling	15 s	head kidney	↑IL-1β
Machado et al.	Solea senegalensis	water acidification	4 h	head kidney	no change in IL-1β
Hoseini et al.	Cyprinus carpio	anesthesia	460 s	head kidney	↓TNF-α, IL-1β
Gao et al.	Paralichthys olivaceus	hyperosmotic	20 min	head kidney, spleen, liver	↑MHC Iα, MHC IIα, CD4–1, CD8α
Magouz et al.	Oreochromis niloticus	ammonia	6 h	liver	↑TNF-α, IL-1β, IL-8
Qian et al.	Micropterus salmoides	lead exposure	96 h	liver	↑C1-C9, MHC I, MHC II
Wiseman et al.	Oncorhynchus mykiss	handling	3 min	liver	↑TNF-α, MHC II
Ammar et al.	Salmo salar	varied (environmental)	10 min	spleen	↑lysozyme G, eosinophil peroxidase, IgM
Su et al.	Scatophagus argus	hyperosmotic & hypoosmotic	1–15 h	renal masses	↑ TNF-α, IL-1β, IL-6, SOCS-1

Hypoxia is a stressor of great relevance for fish, a study subjected Atlantic salmon on different experimental diets to 90-minutes of hypoxia and subsequent re-oxygenation for 48 hours, following vaccination for infectious pancreatic necrosis virus (IPNV) [61]. Kidney antiviral related immune transcripts did not vary in a biologically significant manner between normoxic and hypoxic cohorts on the control diet. However, animals supplemented with a β−1,3/1,6-glucan plus astaxanthin enriched diet demonstrated inhibition of antiviral related immune transcripts in the hypoxic cohort compared to the normoxic cohort [61]. These results highlighted the importance of diet and feed as considerations that can influence results in acute stress-immunity studies.

Lastly, wider transcriptomic studies have also been utilized to elucidate differences between acute and chronic stress mediated immunomodulation. Uren et al. [76] treated Atlantic salmon with acute cold shock, in contrast with chronic stress in the form of absent environmental enrichments. A post-stress LPS challenge demonstrated that the transcriptomic gill profile of the acute stressed cohort differentially expressed the same genes that were differentially expressed in the double negative control cohort, but to a greater magnitude, indicating an immune-stimulated state. Whereas the chronically stressed cohort was overall depressive for the LPS-induced genes, over 200 genes were less dramatically regulated compared to the control group in response to the LPS challenge, many of which were related to the pro-inflammatory response and LPS response Uren et al. [76].

The discrepancies described between the trends of these seemingly contrasting studies can be caused by a variety of different factors in the respective study systems. Additionally, many of these studies did not co-assess stress levels with immune changes in the experimental animals to control for the presence of a stress reaction and requisite response. While many of the referenced results suggest an immune activated state following acute stress, these uncertainties ultimately make it difficult to responsibly draw certain conclusions about the immune transcriptomic response to acute stress, especially the larger systematic impacts these enhancements or suppressions have on the animals.

## 6 Temporal transition between acute and chronic stress in the context of immunomodulation

Relative to acute stressors, the immunomodulating nature of chronic stressors has been clearly demonstrated [87]. In this review, we tentatively suggest that short-term stress mediated immunomodulation is notably different than immunomodulation mediated by chronic stress. This section attempts to provide further insight into the temporal transition between acute and chronic stress induced immunomodulation.

An *in vitro* cortisol-based study in liver tissues demonstrated that *SOCS-1* and *suppressor of cytokine signaling 2* (*SOCS-2*) transcripts were upregulated in response to cortisol at 24-hours, correlating with a measured decrease in *IL-6* and *IL-8* liver transcript levels at the same timepoint. However, in comparison, upregulation was not observed for *SOCS-1* and *SOCS-2* at the 8-hour time point [54]. In these experimental parameters, the results suggest that cortisol mediated upregulation of these immune suppressors occur between 8- and 24-hours. Meanwhile, it is important to consider that the *in vitro* nature of the study precludes consideration of other stress hormones that could also be involved in this interaction in a natural *in vivo* setting.

A study that investigated the cellular profiles of common carp subjected to a 3-hour drop in temperature demonstrated an increase in circulating B cells and granulocytes. In contrast, while head kidney populations of B cells were increased, while granulocytes were decreased [26]. These results were also hinted at from an earlier study of Coho salmon (*Oncorhynchus kisutch*) where leukocyte numbers fluctuated significantly in various tissues after a 1-day acute stress and returned to pre-stress levels in the respective tissues 3-days post stress [51]. Holistically, these findings suggest a redistribution of immune cells in fish following acute stress and a reversion of those movements thereafter, a mechanism perhaps conserved to what was discovered in mammals, albeit with a faster timescale [20].

Additionally, a zebrafish (*Danio rerio*) study where fish was exposed to a chemical pollutant for 24-, 48-, and 72-hours demonstrated upregulations for *IL-8*, and *TNF-α* transcripts at both 24- and 48-hours, but the same transcripts were downregulated at 72-hours [40]. A similarly designed study of acute stress in goldfish (*Carassius auratus*) contrasting a 24-hour schedule with a 72-hour schedule. Interestingly, the investigators found an improvement in plasma lysozyme and complement activity in the 24-hour stress cohort, and a decrease in those same parameters in the 72-hour cohort [27]. These results could be interpreted as a sign of transitioning between acute-stress and chronic-stress immunomodulation. In which case, the results present the idea of a 72-hour stress period as a possible benchmark for the transition period between acute and chronic stress.

However, this proposed timeframe would not be congruent with mammalian studies where research has demonstrated that while a 1-minute handling stressor can induce an increase in circulating mitogen-induced proliferation of T and B cells, a 2-hour immobilization stressor can induce a decrease in the same parameters [60]. These results demonstrate that a stressor as short as 2-hours can start displaying signs immunosuppression, a duration typically viewed as acute or short-term. Still, the lack of literature on the specific topic in fish and the contradictory findings between models make it difficult to propose a specific time range. While this information can be elucidated with more targeted studies, Research of this nature has not yet been conducted in the fish model to the best of our knowledge.

Ultimately, the practicality of establishing a time frame for the transition between the two stress types may be minimal, with involvement from factors like type and severity of the stressor that can also affect the immunomodulation. Although the information in fish is scarce, mammalian studies have indicated a stress duration of minutes to hours can significantly stimulate the immune system. Therefore, for pragmatic purposes, researchers looking to either study acute stress immunomodulation or to use acute stress in an applicable manner to stimulate immune activity, it may be beneficial to err on the side of a shorter rather than a longer acute stress protocol.

## 7 Limitations of the field and state of current knowledge

The nature of these stress-immunity studies involves many specific considerations regarding experimental design, such as intensity of stress [37], developmental stage of experimental animals [28], the diet of animals [61], type of pathogenic pressure [16], and species of experimental animals [5,^6^,44], among other factors. With each compounding factor, they affect the result of the experiment, and it becomes increasingly difficult to compare results between different studies in the same field with varying experimental design parameters. In comparison, the current field of related studies heavily focus on looking at short-term innate, humoral, and transcript level changes in the subjects. While studies on the effects of acute stress on adaptive immunity and studies on long-term immunological changes are relatively scarce.

Current experimental designs of studies in this field are also a limiting factor for the current level of elucidated knowledge. Many of the studies discussed in this review employed typical stressors in an acute time course, but did not specifically quantify or measure the stress response as verification. Additionally, of the performed acute stress treatments, most studies rarely included a pathogen challenge aspect to the design. Lastly, *in vitro* studies in this field also have their limitations, especially considering immune responses to acute stressors involve signaling from more than one type of stress hormone.

An example of an optimal study to elucidate the most significant impact of acute stress on fish immunity would be a focused, *in vivo* study of a combination of acute stress, and live pathogen challenge, with analysis of more functional parameters like mortality rates, in conjunction with expression regulation analysis to demonstrate the potential fitness consequences of acute stress immunomodulation. However, to date, studies with this level of focus and detail on acute stress-immunity are scarce to none. With so many differences between the factors employed in the experimental methods of these studies, and studies that have presented contrasting data [48], it is difficult to accurately suggest the cause(s) for discrepancies between these results. More research needs to be conducted to further elucidate and connect the presently observed acute stress-mediated regulation changes to functional changes in immune performance and activity.

## 8 Conclusions

Overall, the currently available literature's depiction of the differences that the effects acute stress and chronic stress have on immunity is congruent with the trends observed in mammalian models. Although there are many results suggesting that acute stress has immunoenhancing effects, both from direct measurements of innate immune parameters and transcriptional analysis, the contrasting data, as well as the differing factors between the study designs employed, makes it difficult to conclusively state that acute stress has a definitive immunoenhancing effect in fish. However, the non-cortisol stress hormone studies have demonstrated a varying array of effects on the immune system that are neutral or immunoenhancing. Similarly, there is enough evidence to suggest that the acute stress response mediates the fish immune system in a less suppressive way than the effects seen during chronic stress. In the future, more focused research has to be conducted to further and more confidently elucidate specific relationships between the acute stress response and the immune response in fish.

## Declaration of Competing Interest

The authors declare that they have no known competing financial interests or personal relationships that could have appeared to influence the work reported in this paper.
